# Health promotion partnership to promote physical activity in Swedish children with ASD and ADHD

**DOI:** 10.1093/heapro/daac169

**Published:** 2022-12-14

**Authors:** Marie Lydell, Lars Kristén, Maria Nyholm

**Affiliations:** School of Health and Welfare, Halmstad University, Halmstad, Sweden; School of Health and Welfare, Halmstad University, Halmstad, Sweden; School of Health and Welfare, Halmstad University, Halmstad, Sweden

**Keywords:** qualitative methods, physical activity, children with ASD and ADHD, health promotion partnership

## Abstract

Children with autism spectrum disorder (ASD) or attention-deficit/hyperactivity disorder (ADHD) have a higher risk of inactivity, and efforts to promote physical activity among this population have been limited. Physical activity on prescription (PAP) may be a suitable tool for motivating participation in physical activity among children with these diagnoses. However, PAP calls for synergy and partnership between health care and other sectors of the community. The aim of this study was to describe a health promotion partnership for physical activity targeting children with ASD or ADHD. Data were obtained through individual interviews with professionals at CAP (*n* = 11) and three focus-group interviews with coaches from local sports clubs. We used the Bergen Model of Collaborative Functioning as the theoretical framework and used qualitative content analysis as the method of analysis to study partnerships between professionals from the Child and Adolescent Psychiatry outpatient clinic (CAP) and coaches from local sport clubs. The findings demonstrate that the partnerships included both positive and negative processes. Although the two partners shared values regarding the project, such as working for a good cause for the children and seeing the potential in the collaboration, there were doubts about sharing common resources and uncertainties about the sustainability of the PAP project. Challenges remain and further research is needed into developing, monitoring and evaluating health promotion partnerships when promoting physical activity for all.

## INTRODUCTION

A sedentary lifestyle is a major public health issue because it contributes to cardio-metabolic risk factors, decreases quality of life and negatively affects cognitive functioning and weight status (Tremblay *et al.*, 2011; Wu *et al.*, 2017). The World Health Organisation (WHO) recommends at least 60 min of moderate to vigorous-intensity physical activity per day for all children aged between 5 and 17 years, but challenges exist in implementing these recommendations in all children (WHO, 2020).

Children diagnosed with autism spectrum disorder (ASD) or attention-deficit/hyperactivity disorder (ADHD) have a higher risk of inactivity, and efforts to promote physical activity among children with these diagnoses have been limited (Murphy and Carbone, 2008; Bandini *et al.*, 2013; Ng *et al.*, 2017). Regular physical activity, regardless of the specific activity, has been found to have a positive effect on executive functions and attentional control in children with ADHD (Gapin and Etnier, 2012; Ziereis and Jansen, 2015; Christensen *et al.*, 2019). Physical activity on prescription (PAP) is one strategy for promoting physical activity among children with these diagnoses, but PAP calls for collaborative partnership at the community level (Ludyga *et al.*, 2020). Health promotion partnerships include arrangements in which people and organizations, governments, foundations and NGOs are merged together to promote health and to create the synergies that are required to accomplish the goals of the health promotion project or strategies (Weiss *et al.*, 2002; Corbin and Mittelmark, 2008; Corbin, 2017). However, building up such partnerships takes time and demands resources, and the partnerships are problematic to manage (Kreuter *et al.*, 2000; Weiss *et al.*, 2002).

### Theoretical framework

Already in 1997 at the Fourth International Conference on Health Promotion held in Jakarta, Indonesia, effective health promotion and partnership was put at the forefront: ‘Cooperation is essential; this requires the creation of new partnerships for health, on an equal footing, between the different sectors at all levels of governance in societies’ [(WHO, 1998), p. 60]. In the literature, partnership has been recognized as a collaborative working relationship that contributes to partners achieving more together than they could on their own, and it has been suggested that partnerships can contribute to local organizations working together to implement policies and to address the needs in the community (Corbin and Mittelmark, 2008; Corbin *et al.*, 2018; Stibbe *et al.*, 2019). Partnership synergy has been suggested to be an indicator of a successful collaborative process, but synergy in partnerships has been shown to be difficult to achieve (Lasker *et al.*, 2001; Butterfoss and Francisco, 2004; Corbin and Mittelmark, 2008). Lasker *et al.* have identified five basic elements that have an impact on partnerships and the ability to achieve synergy, namely resources, partner characteristics, the relationships between the partners and the external environment (Lasker *et al.*, 2001). Lasker and Weiss have further developed three practical-level factors to create synergy in partnerships, namely who is involved in the partnership, how they are involved and how well the leadership and management of the partnership support the interactions of the partners (Lasker and Weiss, 2003). The Bergen Model of Collaborative Functioning (BMCF) has been used as a systematic guide for practice for partnership, with input, throughput (collaboration), output and feedback factors (Corbin and Mittelmark, 2008; Corbin *et al.*, 2018). The inputs to partnerships are partners’ resources, finances and missions, and these contribute to the dynamics of the partnerships. The throughput factor is the collaborative process and includes leadership, communication, roles, structure, power, trust and funding/partner balance, and the output of the models includes additive and antagonistic results and synergies (Corbin and Mittelmark, 2008; Corbin *et al.*, 2018). Therefore, the aim of this study was to describe a health promotion partnership for physical activity targeting children with ASD or ADHD. In using BMCF as the theoretical framework, we analyse the partnership between professionals from the Child and Adolescent Psychiatry outpatient clinic (CAP) and coaches from local sport clubs.

## METHODS

### Settings

#### Health promotion partnership

The partnership was built on a CAP outpatient clinic, the Regional Sport Federation and local sport clubs. The outpatient clinic is one of four clinics in a region in southwestern Sweden, and the clinic is situated in the capital of the region (with about 70 000 habitants). The professionals at CAP received information about the PAP project, and two assistant nurses were chosen to be the contacts for the project at the CAP. The Regional Sport Federation works to support the sports movement in the region through public education and sports association development and by collaborating with and giving support to all sport clubs in the region. In the PAP project, a coordinator was involved from the Regional Sport Federation. Local sport clubs received a request from the coordinator to participate in the PAP project, and sport clubs that were involved in the PAP project received financial support from the Regional Sport Federation. Coaches from the local sport clubs led the activity and offered an adaption within the regular activities for children with ASD or ADHD. The coaches were performing the activities voluntarily and received information about the children with ASD and ADHD from the Regional Sport Federation and the CAP professionals. Children and their parents who were visiting the outpatient clinic for a regular check-up were offered PAP. They could choose between sports at different local sport clubs, the frequency per week and the intensity. The offer lasted for 1 year at a fee of 10 euros. The CAP professionals wrote the prescription and sent it to a coordinator at the Regional Sport Federation who in turn contacted the local sport clubs and helped the child to get started with physical activity.

### Design and methodological approach

A qualitative design was used to describe the views of the professionals at CAP and the local sports coaches regarding the collaborative work. Data were collected through individual and focus-group interviews, and open-ended questions were used together with probing questions (Hsieh and Shannon, 2005). Data were analysed via qualitative content analysis with an inductive approach (Graneheim and Lundman, 2004; Graneheim *et al.*, 2017; Lindgren *et al.*, 2020).

### Interviews and data collection

#### Informants: professionals at CAP

All professionals involved in the PAP intervention (*n* = 11) were invited for an interview. Ten women and one man, with a mean age of 47 years (31–61 years), participated. One was a manager, four were nurses, two were psychologists, two were social workers, one was an occupational therapist and one was an assistant nurse. Participants had worked at CAP for a mean of 9 years (2–27 years). Data from the individual interviews were collected in August and September 2016 by all three authors (not employed at CAP). The interviews were conducted at the participants’ workplaces at CAP and lasted 30–45 min. An interview guide was used to facilitate the interviews. The interview guide included initial questions and key questions about the project, with the addition of probing questions to elicit further information. Questions included: What are your experiences in prescribing physical activities to children with ASD and ADHD? What are your expectations of the collaboration between CAP and a local sports club? What support do you need for the collaboration? Do you have any other thoughts regarding the project? During the interviews, the participants were encouraged to provide more detailed statements relating to their experience by responding to follow-up questions such as: How do you mean? Can you explain further? What more can you tell me? The interviews were recorded digitally, and observational field notes were written and subsequently transcribed verbatim.

#### Informants: coaches from local sports clubs

Coaches from different local sports clubs were informed and invited to participate in the study, both orally and in writing, in a meeting at the Regional Sport Federation. All in all, 15 coaches were invited to participate in focus-group interviews, and 10 coaches chose to attend one of three focus groups. The number of participants in the focus groups ranged from three to four coaches, representing a variety of sporting disciplines, including archery (*n* = 1), bowling (*n* = 2), table tennis (*n* = 1), rowing (*n* = 1), shooting (*n* = 2), climbing (*n* = 1), Jiu jitsu (*n* = 1) and skateboarding (*n* = 1). The data from the focus groups were collected in August and December 2016 by a moderator and an observer (L.K. and M.L.) who were not connected to any of the clubs. One of the focus-group interviews was conducted at a conference room at Halmstad University and two at the Regional Sport Federation. The interviews lasted 45–60 min.

An interview guide was used to facilitate the focus-group interviews. The interview guide included initial questions and key questions about the project with the addition of probing questions to obtain further information. Questions included: What are your experiences in supporting children with ASD and ADHD? How do you view the collaboration with CAP? What support do you need for the collaboration with CAP and coordinator from the Federation? Do you have any other thoughts regarding the PAP project? The coaches were encouraged to develop their answers, and at the end of the interview they were asked if they had anything more to add. The interviews were digitally recorded and subsequently transcribed verbatim.

### Data analysis

#### Focus groups and interviews

We analysed the data via qualitative content analysis in accordance with Graneheim and Lundman’s method (Graneheim and Lundman, 2004; Graneheim *et al.*, 2017). Both the individual and focus-group interviews were transcribed verbatim, and the transcripts from the interviews were read thoroughly and carefully several times by all authors. This process aimed both to understand what was expressed by each and every informant and to gain a sense of the whole (Granheim and Lundman, 2004; Hsieh and Shannon, 2005). The interviews were then analysed (see Table 1) to identify statements that represented the informants’ views of the collaborative work, and units of meaning were identified and then condensed in such a way that they still retained their content (Graneheim and Lundman, 2004; Malterud, 2012; Graneheim *et al.*, 2017). The condensed units of meaning were coded, and codes with similar content were sorted into sub-themes. As an example, codes describing a positive attitude towards physical activity, the negative sports world of today, children being excluded from sport, children being included in sport, children gaining access to sport and children being allowed to try things out together formed the sub-theme ‘working for a good cause’. Sub-themes with similar content were interpreted and abstracted into three main overarching themes (Table 1).

**Table 1:** Example of the analytical process of the study, moving from text to sub-theme and main theme

Meanings units	Sub-theme	Main overarching theme
….the sports movement still tries in every way to get out whatever it is, the young people in one, yes in society’s sporting community (*focus group, sport coach*)	Working for a common cause	Shared values
….that they get into this with cooperation and sports and what is it called, club feeling or teamwork and for us it is extremely important that everyone should be able to participate, and for as long as possible (*focus groups, sport coach*)	Working for a common cause	Shared values
….if we haven’t received that right away, which I think would be a good thing, that is if we received any information about the young people, communicate with those who have made sure that they come out to training (*focus groups, sport coach*)	Communication with each other	Barriers to communication
….I don’t care, how I get the information. I need to know more about the childrens’ diagnosis (*focus groups, sport coach*)	Communication with each other	Barriers to communication
…. you need to have committed people involved, otherwise there will be uncertainty in the project (*interview with psychologist*)	Feelings of uncertainty	Triggers of doubt
….that some who live outside the city do not have such easy access to it. And it is also perhaps a question of resources for them to be able to come to town and then they cannot participate (*interview with nurse*)	Feelings of uncertainty	Triggers of doubt

## FINDINGS

Three themes—namely shared values, barriers to communication and triggers of doubt—were formulated from professionals at CAP and sports coaches’ descriptions of facilitating the partnership in practice. Both professionals at CAP and coaches shared the same values and vision regarding the importance of increasing physical activity among children with ASD and ADHD in the community. The sharing created a kind of positive dynamic for the partnership and the project. However, both partners encountered obstacles in the collaborative working process as barriers to communication with the parents and with each other. The complexity of the collaborative work was revealed, and doubts about the partnership and the PAP project were raised.

### Shared values

The CAP professionals and sports coaches held shared values regarding the contribution of physical activity to wellbeing and had a similar vision regarding the importance for children, and particularly for children with ASD and ADHD, to be physically active. For both partners, the partnership was about working for the good of the children, and both partners saw the potential of the collaborative work.

### Working for a good cause

The positive influence of physical activity on physical and social wellbeing among the children with ASD and ADHD was discussed by both the CAP professionals and the coaches. Both partners considered it important and a good cause to improve opportunities for the children to participate in sports because children with these diagnoses often have difficulties participating in such activities or in becoming a member of a sports club.

Physical activity is important, and many children become sedentary, sitting at home, do not go out—they have difficulty in their social life, they can be social but they have difficulties when there are several different people around, when it gets a little loud and so on… (female professional at CAP for 5 years)

The professionals at CAP discussed the barriers to the children becoming part of a sports club and participating in regular training sessions and in team sports. Some of the coaches said that if the PAP project did not exist, the children with ASD and ADHD would have had no opportunity to take part in sports training. The coaches referred to the element of competition as a potentially negative side to sports at a young age and concluded that sport should be for all.

Free exercises in sports are missing, in football and so on; not everything should be about playing matches, racing and things like that… (focus group 1)

### Seeing the potential with the partnership

The CAP workers expressed the opinion that physical activity and the PAP project could become the profile of the clinic. Offering physical activity to children was perceived by the professionals at CAP as a pleasant experience, as it was something different to add to the medical therapy. The offer of physical activity was very well received by parents, some of whom had had negative experiences with leisure-time activities for their children in the past. The existence of bad attitudes in sports clubs was confirmed by the coaches in their discussion of the social culture of today’s clubs and the lack of time and space for children with ASD and ADHD to take part in sports. The coaches found that the collaboration with CAP had some other relative advantages, such as gaining new club members, and they felt that the PAP project highlighted the ‘soul of sports’, demonstrating that everyone can participate in sport. This ‘soul’ was described in terms of being engaged socially as well as simply participating.

We got six new members, who think this is great, and our other members thought, yes they are nice, they are funny, those guys. (focus group 1)

Another perceived advantage of the PAP project identified by professionals from CAP was that it helped to offer the parents and their children a more holistic approach at check-ups. Basic issues such as daily routines, sleeping problems and social isolation were discussed together with participation in physical activity.

If the children express that it’s hard to find something that motivates them, as they get easily bored, then I usually talk about the PAP project, which provides an opportunity to try out activities. (female professional at CAP for 3 years)

### Barriers to communication

The professionals at CAP and the sport coaches described challenges in interactions with parents and in communication with each other, and these constituted barriers to communication in the practice of the PAP project.

### Experiencing challenges in interactions with parents

Both the professionals at CAP and the sports coaches found that it was a challenge to interact with parents about the PAP project and the procedures involved. The professionals at CAP sometimes felt hesitant about informing the parents about the project at check-ups because they were aware that in some cases the children could be resistant to physical activity. Professionals at CAP were also aware of the parents’ already heavy workload due to their child’s needs and care:

…when you are a parent and come home to your everyday life, then it becomes a completely different matter, you need transport that works and the time needs to be right and then the rest that needs to be taken care of as a parent. (female professional at CAP for 2 years)

The sports coaches experienced that the parents of children with ASD and ADHD were more cautious and had more questions about the activities compared to other parents. The parents also liked to be present in the sports hall during training, and in some cases interrupted activities. Some parents were pleasantly surprised by their child’s abilities in the sport and wanted to film them during training, which the coaches experienced as disruptive.

… I had put coffee and gingerbread on the table for the parents because I did not want them to be in the sports hall. They could all sit down and have a cup of coffee … because I wanted the children to myself … (focus group 3)

### Communicating with each other

The collaborative work was designed with a coordinator as a communication link between CAP and the sports clubs. The coordinator had good knowledge about both organizations and was perceived as important for guiding and supporting the collaboration between the two organizations and their cultures. The professionals and coaches spoke of both advantages and disadvantages of having the coordinator as a connector between CAP and the sports clubs. Advantages included feeling secure about the collaborative work, as in having the right information about the PAP project. On the other hand, there was a lack of knowledge about the other partner because there was no direct contact, and this created a certain level of suspicion. The professionals at CAP stated that they wanted more knowledge and information about the sports coaches and about the physical activities that were being offered to the children. They also wanted to know whether the coaches had any training in supporting children with ASD or ADHD because some of the children were prone to aggressive behaviour.

…no, above all, I think [it’s important] to have coaches who understand them and their diagnoses and to be able to help them to behave in a good way. It is not just ADHD, but there is also oppositional defiant disorder, for example. We have many girls and boys who are angry and who cannot tolerate anything … (female professional at CAP for 5 years)

Both the professionals at CAP and the sports coaches expressed a need for bridging the gap between the two cultures through dialogue, with the opportunity to share experiences and knowledge, in order to create a more solid collaboration. Coaches suggested that sharing experiences between coaches was important for the PAP project, but that there was also a need for a line of direct contact to the professionals at CAP.

Someone you can call and talk about what had happened. Where I could ask what they think about this and what I should do in this situation and then they perhaps cannot always answer, or are not able to answer, but just to give me some tips or advice in any case. (focus group 3)

Both the professionals at CAP and the sports coaches described communication with the other partner as being characterized by a certain amount of suspicion because they did not know very much about each other. They suggested a system for providing constant feedback, such as whether the child enjoyed the activity and whether the child was matched to the right sport and at the right level.

### Triggers of doubt

Both partners had doubts about the sustainability of using common nonfinancial resources in the PAP project, such as the sport club’s reliance on voluntary work. The lack of trust between the partners and the resistance to being a part of the project also contributed to the triggering of doubts about the collaborative work.

#### Using common resources

Both partners discussed resources in terms of responsibilities, organization of the PAP project and funding. The professionals at CAP and the sports coaches instinctively spoke about the PAP project’s resources, and they perceived that the project was only temporary and therefore had concerns about what would happen to the collaborative work if support and funding was stopped, for example, the funding for the coordinator. The sports coaches said that without some amount of financing and support, offering sports activities to children with ASD and ADHD would be difficult. They also drew attention to a lack of personnel resources because there was a shortage of coaches in every sports club. Funds for facilities were also a worry, and one club even needed a new sports hall and various sports equipment.

… that’s, it’s not possible because it is voluntary work, the money is needed, and yes, we need a contribution from the government in some way … (focus group 2)

The professionals at CAP felt that they were responsible for offering physical activity to the children and their parents, and they pointed out the need for support and resources from management and administration at the clinic. Another resource problem was that due to tight schedules and time pressures at the clinic, information about the PAP intervention was easily forgotten, and information about the project was sometimes simply delivered to parents in writing without any oral explanation.

… it has happened that you have not thought about it or just forget about it … (female professional at CAP for 8 years)

#### Feelings of uncertainty

Both the professionals at CAP and the sports coaches experienced feelings of uncertainty regarding the practical work in the collaboration. The professionals at CAP felt uncertain about CAP as an arena for promoting the PAP project because the physical activities were performed outside the health care setting, and questions arose about who held responsibility for the child. Instead, the children’s schools were suggested to be a more suitable arena for promoting the project and for participating in physical activity. Further, some CAP workers were hesitant to take part in the project and to inform the parents about it because many of them were already working within several different projects.

… my first thought was that … gosh, again another project to talk about with the parents… I felt a bit like that and then you must do it in a certain way … I just felt that I did not want to be involved in more things, but I know that this is good for the children and their families… (female professional at CAP for fifteen years)

The sport coaches expressed their own fears and uncertainties, for example, in relation to meeting, reaching out to and interacting with the children. However, fears and expectations changed over time due to positive feedback from the parents. When the interactions succeeded, the coaches experienced this as a personal development.

I expected it to be worse than it was. That there would be many more outbreaks and conflicts than there have been. There have been some who have become a bit angry and like that, we just let them be… (focus group 1).

## DISCUSSION

Promoting physical activity in children with ASD or ADHD demands resources and skills as well as partnership with more than one organization (Christiansen *et al.*, 2019). The purpose of this study was to describe a health promotion partnership for increasing physical activity among children with ASD and ADHD. The findings have implication for the collaborative process between the health care sector and local sport clubs in promoting physical activity.

Partnerships have been discussed in the literature as difficult to manage, and the mechanisms behind failed and successful partnerships are an ongoing debate because most partnerships dissolve within a year (Mitchell and Shortell, 2000; Corbin *et al.*, 2018). However, a key factor for partnership synergy is having agreed-upon goals and a shared vision (Kreuter *et al.*, 2000; Corbin and Mittelmark, 2008; Jones and Barry, 2011). In this study, the partners displayed shared values and visions regarding promoting physical activity in children with ASD and ADHD, and both partners were positive and saw the potential with the partnership. However, this is not a guarantee for a successful collaborative process because doubts and uncertainty about the practical way to connect to the desired goal and outcomes might jeopardize the process (Kreuter *et al.*, 2000; McInnes *et al.*, 2015). Lasker and Weiss discussed the importance of the right leadership and management to be able to bring people together who have no common background of working together (Lasker and Weiss, 2003). The partnership described here involved various partners, and the professionals at CAP and the sport coaches had no experiences in health promotion partnerships. Part of the collaboration was a top–down initiated collaboration, where the professionals at CAP were invited through management at CAP and the sport coaches were invited to participate voluntarily. Partnerships that are initiated through top–down collaboration contribute with difficulties in the relationship between partners (Lasker *et al.*, 2001; Van Meerkerk, 2019).

Trust has been described as a key factor for effective functioning of partnerships, and in health promotion partnerships two levels of trust are functioning, including partners trusting each other and trusting the organization the partners represent (Jones and Barry, 2011). The professionals at CAP described the children’s negative experiences when participating in sports. This so-called bad reputation or past behaviour might contribute to suspicions about the partnerships with the sport coaches, and building up trust between organizations with no clear history of collaboration is difficult, time-consuming and resource intensive (Tooher *et al.*, 2017). To overcome these problems, a coordinator was used in this project to be the contact person for both the professionals at CAP and the sport coaches, thus important dialogues and follow-up meetings about the children might have been missed between the professionals at CAP and the sport coaches.

This health promotion partnership might include parts that are critical to producing synergistic outcomes; however, triggers of doubts were revealed by both partners regarding the sharing of common resources and funding and uncertainties about the sustainability of the PAP project. However, the output could be described according to the BMCF as both antagony and synergy, and Corbin and Mittelmark (Corbin and Mittelmark, 2008) discussed how synergy and antagony can exist at the same time in a partnership. The antagony in the partnership described here was the triggering of doubt among the partners, and the synergy was some children with ASD and ADHD being able to participate in physical activity at a local sport clubs.

### Limitations

All professionals at CAP who were involved in the PAP project participated in the study. Sports coaches from different local sports clubs were also invited to participate, although not all invited sport coaches participated, and it may be that the coaches with the most interest in the project participated in the focus-group interviews. Focus-group interviews might not be the optimal way to collect data from the coaches because their work in the sports clubs was voluntary and the interviews were conducted in their spare time, and this might have influenced the participation rate among the coaches. Individual interviews might be a more flexible way to collect data. Also, the participation rate among the families was low in this study, and a web-designed questionnaire might perhaps be more suited for busy families instead of face-to-face interviews.

The trustworthiness of the content analysis was partly ensured by the fact that the researchers in the team had a good level of knowledge and experience of qualitative methodology (Graneheim and Lundman, 2004). With three different sciences represented (adapted physical activity pedagogy, public health science and physiotherapy), the discussions within the team were interdisciplinary, and inputs and critical comments were used to strengthen the analytic process. All members of the team collaborated at every step of the analysis until final agreement was reached on the sub-themes and themes (Graneheim and Lundman, 2004; Graneheim *et al.*, 2017).

## CONCLUSIONS

This study presents a health promotion partnership between CAP and local sport clubs to promote physical activity in children with ASD and ADHD. The input of the partnership was shared values among the partners in working for a good cause and seeing the potential with the partnership. Thus, both antagony and synergy were seen in the output of the partnership. Antagony in the form of doubts was revealed by both partners, and synergy was demonstrated by the involvement of the children in physical activity at local sport clubs. This study reveals the complexity of research in health promotion partnership in promoting physical activity and in evaluating and measuring how much the collaboration contributes to the impact of the output and its effectiveness in promoting physical activity. However, health promotion partnerships have an important role to play in promoting physical activity for all, and therefore more research is needed to encourage and support and to understand what is and what leads to positive sustainable results in partnerships. Different indicators need to be developed to be able to document the sustainability and effectiveness of partnerships in promoting physical activity for all.
